# Dispersion and shape engineered plasmonic nanosensors

**DOI:** 10.1038/ncomms11331

**Published:** 2016-04-19

**Authors:** Hyeon-Ho Jeong, Andrew G. Mark, Mariana Alarcón-Correa, Insook Kim, Peter Oswald, Tung-Chun Lee, Peer Fischer

**Affiliations:** 1 Max Planck Institute for Intelligent Systems, Heisenbergstrasse 3, 70569 Stuttgart, Germany; 2 Institute of Materials, École Polytechnique Fédérale de Lausanne (EPFL), CH-1015 Lausanne, Switzerland; 3 Institute for Physical Chemistry, University of Stuttgart, Pfaffenwaldring 55, 70569 Stuttgart, Germany; 4 UCL Institute for Materials Discovery and Department of Chemistry, University College London, Christopher Ingold Building, 20 Gordon Street, London WC1H 0AJ, UK

## Abstract

Biosensors based on the localized surface plasmon resonance (LSPR) of individual metallic nanoparticles promise to deliver modular, low-cost sensing with high-detection thresholds. However, they continue to suffer from relatively low sensitivity and figures of merit (FOMs). Herein we introduce the idea of sensitivity enhancement of LSPR sensors through engineering of the material dispersion function. Employing dispersion and shape engineering of chiral nanoparticles leads to remarkable refractive index sensitivities (1,091 nm RIU^−1^ at *λ*=921 nm) and FOMs (>2,800 RIU^−1^). A key feature is that the polarization-dependent extinction of the nanoparticles is now characterized by rich spectral features, including bipolar peaks and nulls, suitable for tracking refractive index changes. This sensing modality offers strong optical contrast even in the presence of highly absorbing media, an important consideration for use in complex biological media with limited transmission. The technique is sensitive to surface-specific binding events which we demonstrate through biotin–avidin surface coupling.

## Abstract (web summary)

Sensors based on localized surface plasmon resonance suffer from low figures of merit. Here, the authors achieve high refractive index sensitivities and figures of merit by introducing a chiral shape and the idea of engineering the material dispersion function.

## Graphical abstract

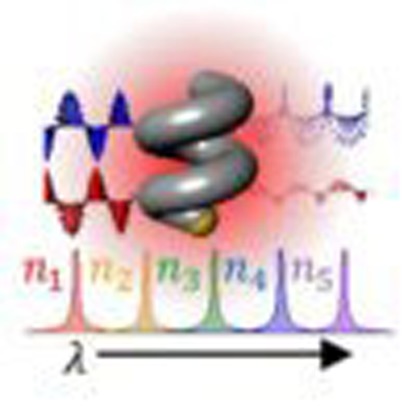

Devices based on surface plasmon resonance (SPR) phenomena detect shifts of the resonance wavelength in response to changes of the refractive index of the medium surrounding the plasmonic material^1^. This may, for instance, be due to biomolecules that bind to the sensor. SPR offers high sensitivities^2^, but requires extended smooth surfaces^3^. In many situations, it would be desirable to have a local sensor for use *in situ* or *in vivo* (for instance within a cell) and here the localized SPR (LSPR) supported by nanostructures offers substantial advantages^4^,^5^,^6^. The short penetration depth of plasmon oscillations into the surrounding fluid makes for small, spatially localized sensors which promise to be effective in a range of biomedical applications^7^,^8^,^9^. However, compared with plasmonic biosensors that utilize extended SPR, which serve as the reference standard for optically addressed sensors, LSPR sensors generally have a reduced sensitivity (*S*_*n*_<1,000 nm RIU^−1^) and a lower figure of merit (FOM) <100 RIU^−1^ (refs 2, 5).

The typical strategy employed to enhance the sensitivity of LSPR nanosensors is to manipulate the aspect ratio of symmetrical nanoparticles, with more elongated particles yielding higher sensitivities^10^,^11^. This approach has been demonstrated with nanorods and nanoprisms that show improved sensitivites^12^,^13^,^14^. On the other hand, the materials selected for such particles have, quite reasonably, been restricted to those with strong plasmonic properties: for example, Ag (ref. 15), Au (ref. 9), Al (ref. 16). This has been driven by the desire for a high figure of merit (FOM) that comes from the low-interband damping, and sharp resonance of pure metals. However, it means that the material dispersion function, a key factor in the sensitivity of LSPR nanosensors, has been limited to the dielectric functions inherent to those pure metals.

Here, we show how dispersion-engineering introduces a new material-based parameter for improving the sensitivity of LSPR sensors. When combined with shape engineering, this leads to extremely high LSPR sensitivities and FOMs, which we report herein. We introduce an analytical model of chiral plasmonic sensing that illustrates the roles of chirality and materials properties for the important sensing characteristics. Based on this understanding we present colloidal nanostructures that are, to the best of our knowledge, the most sensitive LSPR sensors reported to date^12^,^17^. Furthermore, we demonstrate the utility of the engineered particles as surface-sensitive probes for biotin–avidin binding. Our scheme is particularly robust as it is immune to changes in the optical density of the background, and can thus be used in complex environments.

## Results

### Theoretical concept

Plasmon-based LSPR sensors operate by tracking the shift in the resonance peak of the plasmon absorption in response to changes in *n*, the refractive index of the local medium^5^. The resonance condition is met when 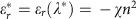, where *λ* is the peak wavelength, *ɛ*_*r*_ is the real part of the dielectric function describing the plasmonic material, and the factor *χ* describes the shape of the particle (for spheres *χ*=2). Ideally, the dispersion of *ɛ*_*r*_ should be such that even very small changes in *n* cause appreciable changes in the resonance wavelength *λ*, that is, large resonance shifts. The sensitivity of the system is defined as^11^


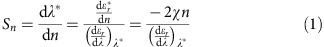


so decreasing the wavelength dependence of the real part of the material's dielectric constant (the denominator) leads to an increase in sensitivity. Here, we engineer the dielectric response of the nanoparticles by alloying a plasmonic material with a weakly dispersive one to yield a negative, but flatter *ɛ*_*r*_. This dramatically increases the sensitivity of any plasmonic sensor (of any shape).

**Figure 1:**
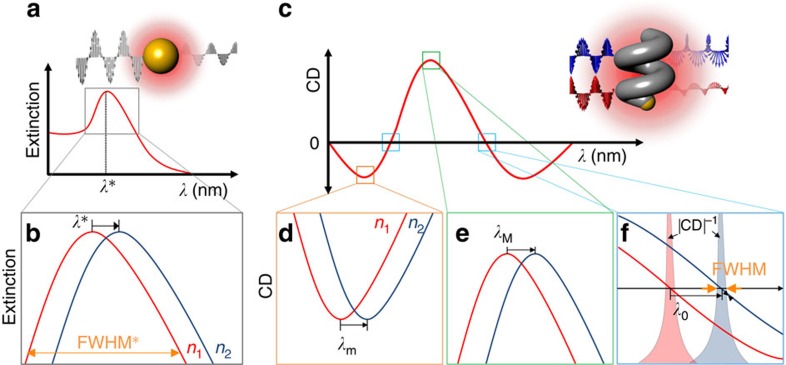
Chiral plasmonic sensing. (**a**,**b**) Schematic view of conventional plasmonic sensing. (**a**) The plasmonic resonance of the metallic nanoparticle as a function of *λ*. Inset shows a conventional plasmonic sensing system where a metallic nanoparticle (here a sphere) interacts with light and generates a detectable absorption peak due to plasmon resonance. (**b**) A zoomed-in region near the peak *λ* showing how it shifts in wavelength as the refractive index of the surrounding medium changes *n*_1_<*n*_2_. (**c**–**f**) Schematic view of polarization-dependent chiral plasmonic sensing. (**c**) The CD spectrum of a plasmonic enantiomer as a function of *λ*. Inset scheme illustrates the interaction of a left-handed nanohelix with circularly polarized light. Three bottom panels indicate the resonance shifts at (**d**) *λ*_m_, (**e**) *λ*_M_ and (**f**) *λ*_0_ where the refractive indices of the surrounding media are varied between *n*_1_ and *n*_2_.

In conjunction with the sensitivity, it is also of interest to consider the accuracy with which a spectral feature can be resolved. For a spectral peak, the standard measure is its full width at half maximum (FWHM). Engineering the real part of the particle's dielectric function^18^ in equation (1) through alloying is generally accompanied by a concomitant increase in the plasmon damping and broadening of the extinction spectrum^19^, and thus suggests that the FOM (=*S*_*n*_/FWHM) is also lowered. This can be addressed by noting that traditional plasmon-based sensors are generally polarization independent, because the particles themselves are highly symmetrical, Fig. 1a. Circular dichroism (CD) offers an alternative to extinction-based sensing techniques^20^,^21^, and CD spectra are typically more feature rich than extinction spectra^22^,^23^, which increases the number of spectral signatures that can be tracked when the local environment changes, Fig. 1b. Crucially, CD spectra are also bipolar, and the crossing points where the signal changes sign are ideal features to track, because of the simplicity of identifying the null point. Earlier work on CD-based sensing made use of magneto-optical modulation of an achiral nanoantenna LSPR to induce ellipticity in the transmitted beam^24^. In this case, the choice of material was dictated by the requirement to induce a magneto-optical response. However, performing CD-based measurements on chiral particles, that exhibit a natural CD, offers the possibility of tuning the material properties independently of the chirality (shape), and we exploit this approach to maximize the LSPR sensitivity (and FOM).

The exact optical responses for such complex nanoparticles are only calculable using numerical methods^25^. However, we introduce the following chiral form of nanoparticle plasmonic absorption^10^,^11^ that captures the key features of extinction in response to left (−) and right (+) circularly polarized light.


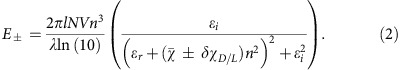


**Figure 2:**
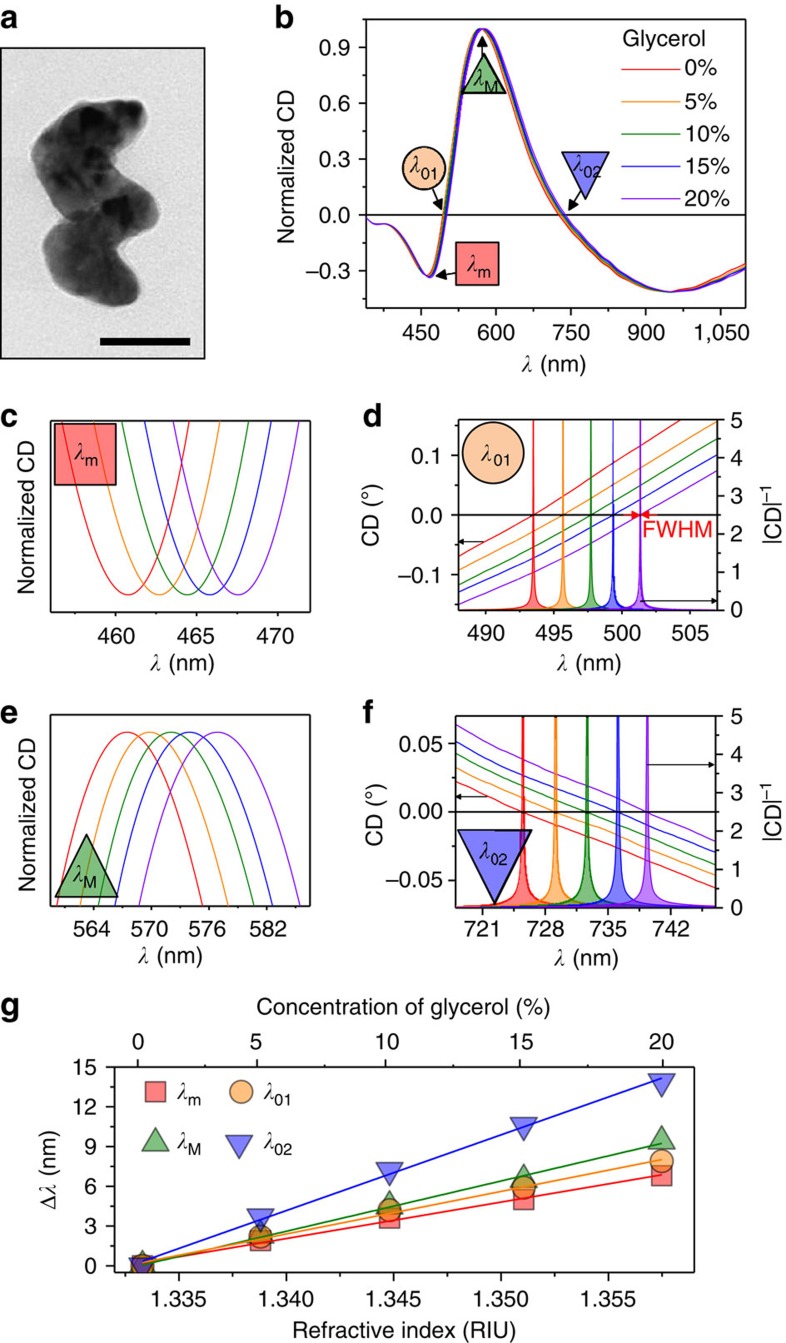
Bulk refractive index sensing. (**a**) TEM image of a single Ag_0.97_Ti_0.03_:*L*_2_ nanohelix (Scale bar, 50 nm). (**b**) CD spectra of colloidal Ag_0.97_Ti_0.03_:*L*_2_ nanohelices in media of five different refractive indices (red: 0%, orange: 5%, green: 10%, blue: 15% and violet: 20% glycerol-water mixtures) over the full spectral range and detailed plots of the resonance shifts at (**c**) *λ*_m_ (red square), (**d**) *λ*_01_ (orange circle), (**e**) *λ*_M_ (green top triangle) and (**f**) *λ*_02_ (blue bottom triangle). For **d** and **f** the filled curves represent |CD|^−1^. (**g**) Wavelength shift, relative to water, of the four spectral features as functions of the glycerol-water concentration (top *x* axis) and its corresponding *n* (bottom *x* axis).

The shape factor now includes an achiral term 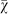 plus a chiral term *δχ*_*D*_=−*δχ*_*L*_ specific to right (D) or left (L) handed enantiomers. The CD is the difference between the extinction for ± polarizations, 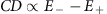. The CD signal is bipolar, and the wavelength at which the extinctions are equal yields a zero-crossing in the spectrum, which serves as a natural point for tracking. Within this formulation the crossover wavelength coincides with the achiral resonance condition 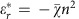, in the limit of small, slowly-varying *ɛ*_*i*_. So the sensitivity of nanohelix sensors follows that of achiral ones. Plotting the reciprocal of the absolute value of the CD (for example, Fig. 2d) gives an intrinsically sharp representation of the crossover point, one whose FWHM is defined by the instrumental resolution *σ* (ref. 24) and 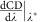, the slope of the CD spectrum at the crossing. This yields a FWHM (see Supplementary Note 1 for details), based on equation (2), of


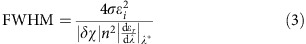


and a FOM of


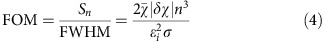


Thus, higher FOM can be achieved by increasing the magnitude of the chiral shape factor *δχ*.

### Fabrication and bulk refractive index sensing

We fabricate a series of hybrid nanohelix sensors using a physical vapour deposition technique that allows precise control of the particles' alloy composition and shape *δχ* (Supplementary Note 2, Supplementary Fig. 1 and Supplementary Table 1)^25^,^26^. The former can be used to engineer the material dielectric function to maximize sensitivity, while the latter is used to adjust the achiral and chiral shape factors to affect sensitivity and introduce a chiroptical response. A typical particle, a two-turn left-handed 128-nm-tall helix composed of 97% Ag and 3%Ti, is shown in Fig. 2a. The small amount of Ti alloying agent improves the helix fidelity over pure Ag (ref. 27). The particles are tested in the form of a colloid suspension whose stability was confirmed through dynamic light scattering (DLS, more details in Supplementary Note 3 and Supplementary Fig. 2). Figure 2b shows CD spectra of the suspensions in glycerol–water mixtures varying from 0 to 20% concentration (refractive indices between 1.333 and 1.357 (ref. 28)). As expected, the CD spectra exhibit multiple features, shown in Fig. 2c–f: a maximum *λ*_M_, a minimum *λ*_m_ and two crossing points *λ*_01_ and *λ*_02_, all of which can be tracked in response to changes in the refractive index of the medium. The wavelength shifts, relative to the pure water reference, are shown in Fig. 2g, and indicate sensitivities *S*_*n*_ of 275, 320, 379 and 571 nm RIU^−1^ for *λ*_m_, *λ*_01_, *λ*_M_ and *λ*_02_, respectively.

### Dispersion and shape engineering of chiral nanoparticles

**Figure 3:**
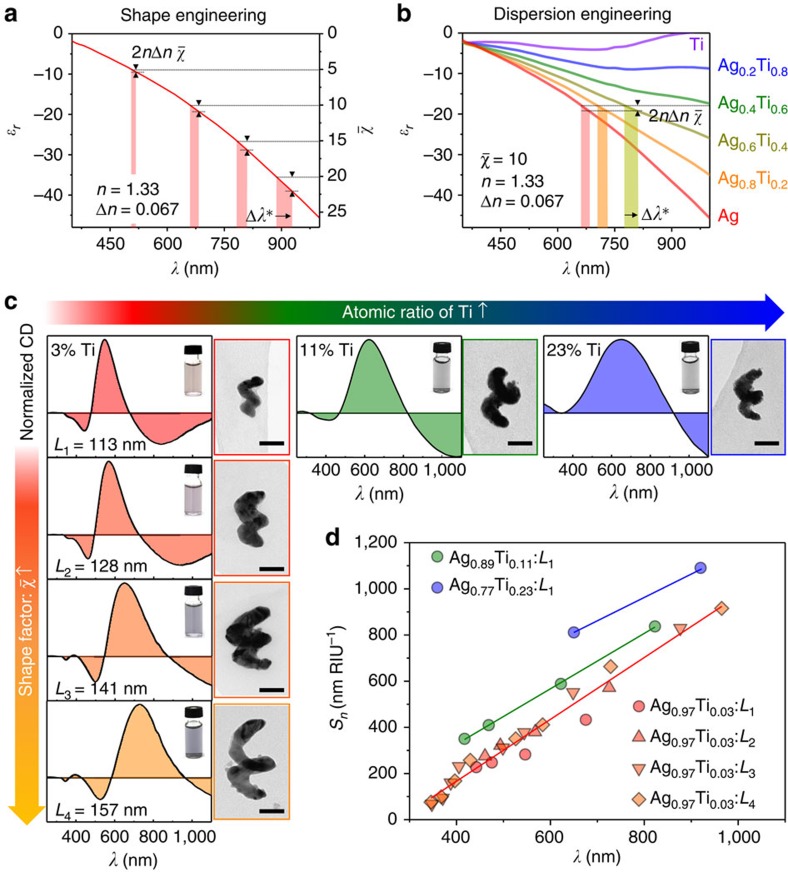
Shape and dispersion engineering of Ag–Ti nanohelices. (**a**) The effect of the achiral shape factor 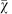 on the wavelength shift Δ*λ* observed for a given change in refractive index Δ*n* (=0.067). (**b**) The effect of *ɛ*_*r*_ on the wavelength shift based on calculated dielectric constant of Ag–Ti alloys with varying Ti composition. As the proportion of Ti increases the real part of the dielectric function becomes flatter, and the resulting wavelength shift increases. (**c**) CD spectra (panel left), TEM images (panel right) and colloidal solutions (each inset) of the grown Ag–Ti nanohelices. The achiral shape factor 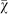 increases moving down the rows, and the atomic ratio of Ti increases moving to the right. (**d**) The measured *S*_*n*_ of the Ag–Ti nanohelices as a function of *λ* at 1.333 RIU (the symbols and colours are different for the 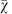 and the atomic ratio of Ti, respectively).

These results demonstrate that features, like *λ*_M_, and *λ*_02_, found at longer wavelengths exhibit greater sensitivity, consistent with equation (1). Since larger values of the achiral shape factor increase the wavelength of the resonance condition, it is possible to increase the sensitivity by growing particles that are more elongated (Fig. 3a). This has been the approach most often pursued in the search for higher sensitivities from extinction-based LSPR sensors^10^,^11^,^12^,^17^. Here we apply the same principle to a chiral sensor, by growing a series of nanohelices with a range of heights, labelled *L*_1_ (113 nm) through *L*_4_ (157 nm). The results, shown in Fig. 3c, confirm that the peak and crossing features of longer structures are red-shifted relative to the smaller ones and that the sensitivity of each feature is directly proportional to its wavelength (Fig. 3d, red points).

However, equation (1) also suggests that the sensitivity can be improved by reducing the wavelength dependence of *ɛ*_*r*_, the real part of the material dielectric function. We do this by exercising control over the structures' Ag–Ti stoichiometry during their growth. Figure 3b shows the effective *ɛ*_*r*_ of Ag–Ti alloys of varying compositions (more details in Supplementary Note 4). As the alloy becomes more Ti-rich, the wavelength shift Δ*λ* increases for a given change in the medium's refractive index, due to a progressive flattening of the material's dielectric function (see also Supplementary Fig. 3 and Supplementary Table 2). The effect of dispersion engineering in practice is illustrated in Fig. 3c for nanohelices having fixed size, but composed of alloys containing 3, 11 and 23% Ti. Higher Ti composition red-shifts the features, and as shown in Fig. 3d, and increases the sensitivity. Indeed, the sensitivity trends show a systematic enhancement for the 11% (green) and 23% (blue) alloy relative to the minimal Ti samples (red; see Supplementary Note 5 and Supplementary Figs 4–9 for detailed plots).

**Figure 4:**
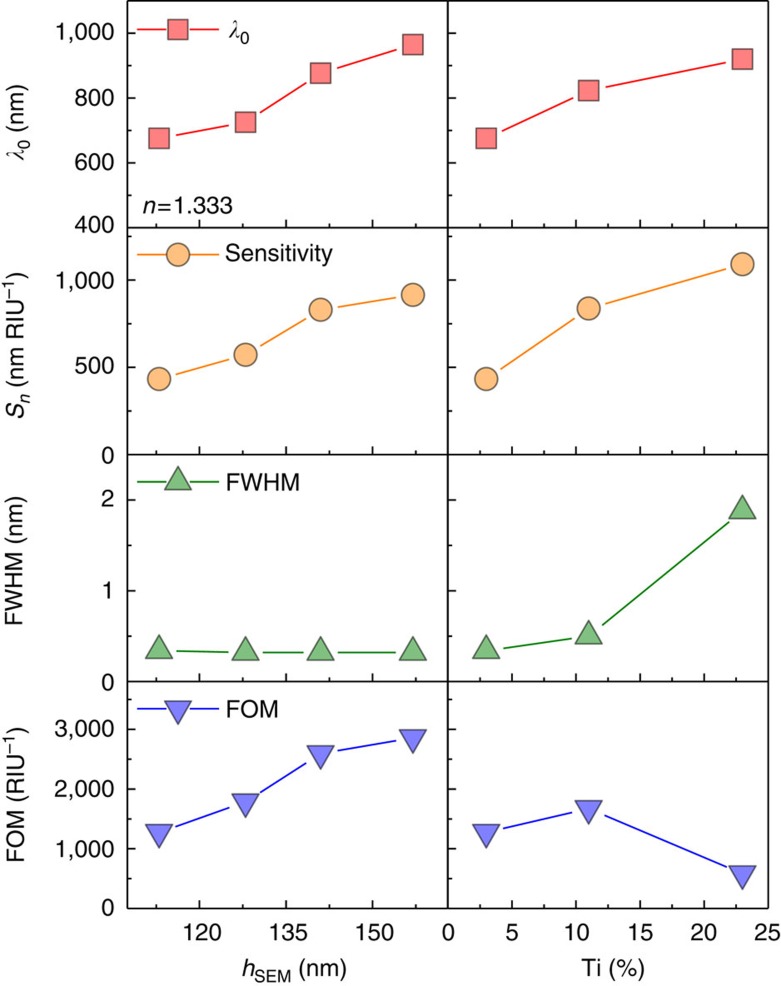
Summary of shape and dispersion engineering of nanohelices. Left panels show the effect of increasing nanohelix length and the right panels show the effect of increasing Ti composition (red square: zero-crossing wavelengths at *n*=1.333, orange circle: refractive index sensitivities, green top triangles: FWHMs and blue bottom triangles: FOMs).

The results are summarized in Fig. 4. The left column in the plots shows the effect of increased nanohelix length on the crossing point wavelength, sensitivity, FWHM and FOM. The null wavelength and sensitivity, both increase as the nanohelices lengthen. However, the FWHM remains unchanged, so that the FOM increases dramatically. The *L*_4_ helix exhibits a FOM=2,859 RIU^−1^, which is larger than what has been previously reported for LSPR-based sensors^13^,^14^,^29^ including those based on a magnetochiral response^24^. Relative to the latter, the improvement comes from a combination of improved sensitivity (∼4 × ) and decreased FWHM (∼5 × ) thanks to a steeper crossing of the CD signal at the null point (Supplementary Note 6 and Supplementary Fig. 10). The right hand column of Fig. 4 illustrates the effect of engineering the dispersion function through the addition of Ti. In this case the increase in sensitivity that comes from flattening the material dispersion function also leads to an increase in the FWHM due to the *ɛ*_*i*_ contribution in equation (3). The net effect is a decrease in the FOM relative to the low-alloy sample. Nevertheless, the flatter dispersion curve does have the effect of increasing the sensitivity beyond what is achievable in the pure metal or the low-alloy sample. Here we observe a sensitivity >1,000 nm RIU^−1^ at a wavelength of 921 nm. For applications where sensitivity is the primary concern, dispersion engineering provides a powerful technique for reaching the highest possible refractive index sensitivities (Supplementary Note 7 and Supplementary Fig. 11).

### Strong optical response in the presence of absorbers

**Figure 5:**
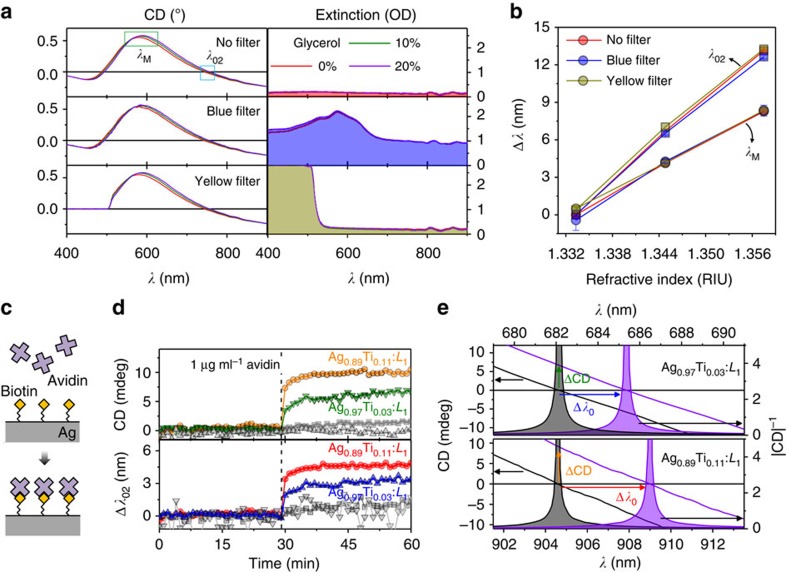
Sensing in absorbing media and Sensing of specific binding events. CD spectra (left panels) and extinction spectra (right panels) of the colloidal Ag_0.97_Ti_0.03_:*L*_3_ nanohelices in the presence of complex absorbing environments (top: no filter, middle: a blue filter, bottom: yellow filter), (**b**) their corresponding wavelength shifts, relative to water, of the two spectral features *(λ*_M_ and *λ*_02_) as functions of the *n* (error bar: s.d.). (**c**) Schematic view of biotin–avidin interaction on the surface of a Ag–Ti nanohelix. (**d**) *In situ* measurements of the biotin–avidin interaction by monitoring the change of CD at the initial *λ*_02_ (upper plot) and the wavelength shift of Δ*λ*_02_ (lower plot) with 1 min intervals. The coloured plots indicate the response of specific binding of biotin–avidin (Ag_0.97_Ti_0.03_:*L*_1_ blue, green; Ag_0.89_Ti_0.11_:*L*_1_ red, yellow) and the grey plots indicate non-specific binding of avidin with Ag–Ti nanohelices (without biotin) in the control group. (**e**) Close-up view of the CD spectra for the two biotinylated nanoparticle systems showing the wavelength shift and CD amplitude increase after avidin introduction.

One appealing characteristic of CD is that it is insensitive to achiral absorbers or scatterers^30^. So unlike traditional extinction measurements, high signal-to-noise CD measurements of a chiral analyte are possible even in the presence of strong achiral absorbers. Figure 5a illustrates such a measurement, where we use a colloidal suspension of Ag_0.97_Ti_0.03_:*L*_3_ nanohelices in water with the addition of absorbers in the form of blue and yellow filters in the optical path. In both cases the maximum absorbance exceeds an optical density of 2 (<0.1% transmission, right panels of Fig. 5a). This introduces small distortions into the CD signal, however, as we have already seen, CD offers several spectral features for sensing, the majority of which are here unperturbed. Notably, since CD-based sensing offers a clearly distinguishable signal even in the presence of absorbance that would be strong enough to obscure optical extinction-based sensing methodologies, Fig. 5b. The same experiment demonstrated with molecular absorbers rhodamine and indigo yield similar results (Supplementary Note 8 and Supplementary Fig. 12).

### Surface-sensitive sensing

Since the evanescent field of the excited plasmon decays rapidly into the medium, LSPR sensors are ideally suited to making surface-sensitive measurements^31^. Here we use our chiral plasmonic nanohelices to sense avidin-binding events. First, the surfaces of our nanohelices (Ag_0.97_Ti_0.03_:*L*_1_ and Ag_0.89_Ti_0.11_:*L*_1_) are functionalized with biotin (see Methods section for details), which acts as a complementary and specific-binding factor for avidin in solution (Fig. 5c). The wavelength of the *λ*_02_ crossing in the CD spectrum (Fig. 5d, lower), and the change in the CD amplitude at the initial crossing wavelength (Fig. 5d, upper) were measured at 1 min intervals. Before the addition of avidin both signals are stable, but they experience a prompt shift on the addition of 1 μg ml^−1^ avidin. For the Ag_0.97_Ti_0.03_:*L*_1_ nanohelices the crossing point Δ*λ*_02_ redshifts by ∼3 nm (blue), and the CD intensity at the initial crossing wavelength increases by 6 mdeg (green). The rise time is ∼5 min. The Ag_0.89_Ti_0.11_:*L*_1_ nanohelices show an even stronger response with shifts of 5 nm (red) and 10 mdeg (yellow) respectively, thanks to the increased sensitivity that comes from the reduced dispersion. Control experiments (grey) where the nanohelices lack biotin functionalization show no distinct shift on the addition of avidin, confirming that the signal is truly surface sensitive and arises from specific binding (see Supplementary Note 9 and Supplementary Figs 13 and 14 for additional details). The two measurement modes suggest alternative sensing schemes for real-time monitoring of binding events: one based on tracking the crossing point wavelength (Fig. 5e, blue and red), and another based on identifying the change in CD intensity at fixed wavelength (Fig. 5e, green and yellow). Both offer a highly sensitive and rapid measurement of the specific binding at surfaces.

## Discussion

We have shown that flattening the dielectric function of plasmonic nanomaterials by alloying results in large LSPR shifts in response to changes in their local environment. If these nanostructures are also chiral, then their polarization-dependent spectra offer additional and sharper spectral features relative to optical extinction-based measurement of achiral particles. These characteristics make chiral plasmonic nanohelices especially suited for sensing applications. Furthermore, the bipolar nature of CD spectra of chiral structures leads to points of zero-crossing, which have very small effective line-widths and thus result in high FOM. Both bulk refractive index changes as well as surface-specific binding events are tracked by the engineered nanocolloidal LSPR sensors with sensitivity (∼1,100 nm RIU^−1^) and FOM (∼2,900 RIU^−1^) higher than any previously reported for plasmonic sensors.

Aside from extremely high-detection sensitivities, these chiral sensors offer unique advantages over existing plasmonic devices. Since they are freely suspended colloids, they naturally suggest themselves as sensors for *in situ* or *in vivo* biological applications, particularly, because of their small size. Since the CD signal is robust against background interference, especially in absorbing media, the sensing scheme reported herein is particularly suited to real biological media, which are typically optically dense. For instance, in the presence of whole blood, our scheme has the potential for real-time measurement of protein corona formation, a vitally important factor for the function of nanoparticles in biological media, but one that remains poorly understood.

## Methods

### Nano glancing angle deposition (nanoGLAD)

The shadow growth technique^32^ was used to grow the three-dimensional nanocolloids in the manner previously reported^25^. First, a hexagonal array of 12 nm Au nanoparticles was prepared by block-copolymer micelle nanolithography^33^. Next, Ag–Ti nanohelices were grown on the array of the Au nanoparticles in a GLAD system based on co-deposition from dual electron-beam evaporators at *T*=90 K with a base pressure of 1 × 10^−6^ mbar. Their alloy stoichiometry was controlled by the deposition rates measured by quartz crystal microbalance (QCM) for each evaporator independently. Particles were grown with lengths (measured by scanning electron microscopy (SEM)) of *L*_1_=113 nm, *L*_2_=128 nm, *L*_3_=141 nm, *L*_4_=157 nm and Ti contents of 3, 11 and 23%. Finally, the grown Ag–Ti nanohelices were lifted off from the wafer by sonicating a piece of sample wafer (0.5–1 cm^2^) in an aqueous solution of 0.15 mM polyvinylpyrrolidone for ∼2 min to prepare a stock solution. To minimize the effect of possible variations in structure and concentration, samples for each set of tests were always drawn from the same stock solution.

### SEM and TEM analysis

The Ag–Ti helices were imaged in SEM (Ultra 55, Zeiss) and transmission electron microscopy (TEM) (CM200, Philips) under the accelerating voltages of 10 and 200 kV, respectively.

### Circular dichroism analysis

CD spectra were obtained with a Jasco J-810 CD spectrometer. All the spectra were measured with 500 nm min^−1^ scan rate in the wavelength range of 300–1,100 nm with 0.1 nm intervals. For selected regions of interest smaller 0.025 nm intervals were used.

### DLS analysis

Colloidal solution (200 μl) of nanohelices was measured using a zeta potential analyser (Zetasizer Nano ZS, Malvern) repeated ∼10 times for 20 min with 2 min intervals. The material property of Ag–Ti nanohelices was fixed to Ag (RI: 0.135 and Absorption: 3.990) and the environmental parameter was matched to literature values for the viscosity, and refractive index of the solution based on the temperature and concentration^28^.

### ICP-OES analysis

Approximately 1 cm^2^ as-grown nanohelices supported on a Si substrate was dissolved into HNO_3_/HF etchant and this solution was analysed by the inductively coupled plasma atomic emission spectroscopy (ICP-OES) (Ciros CCD, Spectro). The material composition of the nanohelices was evaluated by repeating the analysis three times with samples cleaved from different areas of the growth wafer.

### Absorbers

Blue and yellow filters were separately inserted into the optical patch either before or after the sample cuvette. Similarly, 10 μM rhodamine 6G and 100 μM indigo were added to separate samples after acquiring baseline measurements.

### Biotin–avidin binding procedure

The mixture of biotin-Polyethylene glycol (PEG)-SH (25 mg ml^−1^ in 3-(N-morpholino)propanesulfonic acid (MOPS)-DNA buffer of pH7.5) and CH_3_O-PEG-SH (250 mg ml^−1^ in MOPS-DNA buffer of pH7.5) in the ratio of 1:1 (v v^−1^) was drop-cast onto ∼0.5 cm^2^ of as-grown nanohelix substrate and kept under humid condition overnight. For the control group, the nanohelices were exposed to only CH_3_O-PEG-SH. Next, each substrate was immersed in 1 ml of 0.15 mM polyvinylpyrrolidone solution and sonicated to remove the particles from the wafer and suspend them in solution. Finally, for the biotin–avidin interaction, 1 μg ml^−1^ of avidin was injected to the colloidal solution and mixed by pipetting.

## Additional information

**How to cite this article:** Jeong, H.-H. *et al*. Dispersion and shape engineered plasmonic nanosensors. *Nat. Commun.* 7:11331 doi: 10.1038/ncomms11331 (2016).

## Supplementary Material

Supplementary Information Supplementary Figures 1-14, Supplementary Tables 1-2, Supplementary Notes 1-9 and Supplementary References.
