# Supplementary material for: Dispersion and shape engineered plasmonic nanosensors
Source: Nat Commun. 2016 Apr 19;7:11331. doi: 10.1038/ncomms11331 (PMC4838895; doi:10.1038/ncomms11331)
Supplement: Supplementary Information — Supplementary Figures 1-14, Supplementary Tables 1-2, Supplementary Notes 1-9 and Supplementary References. [file ncomms11331-s1.pdf]

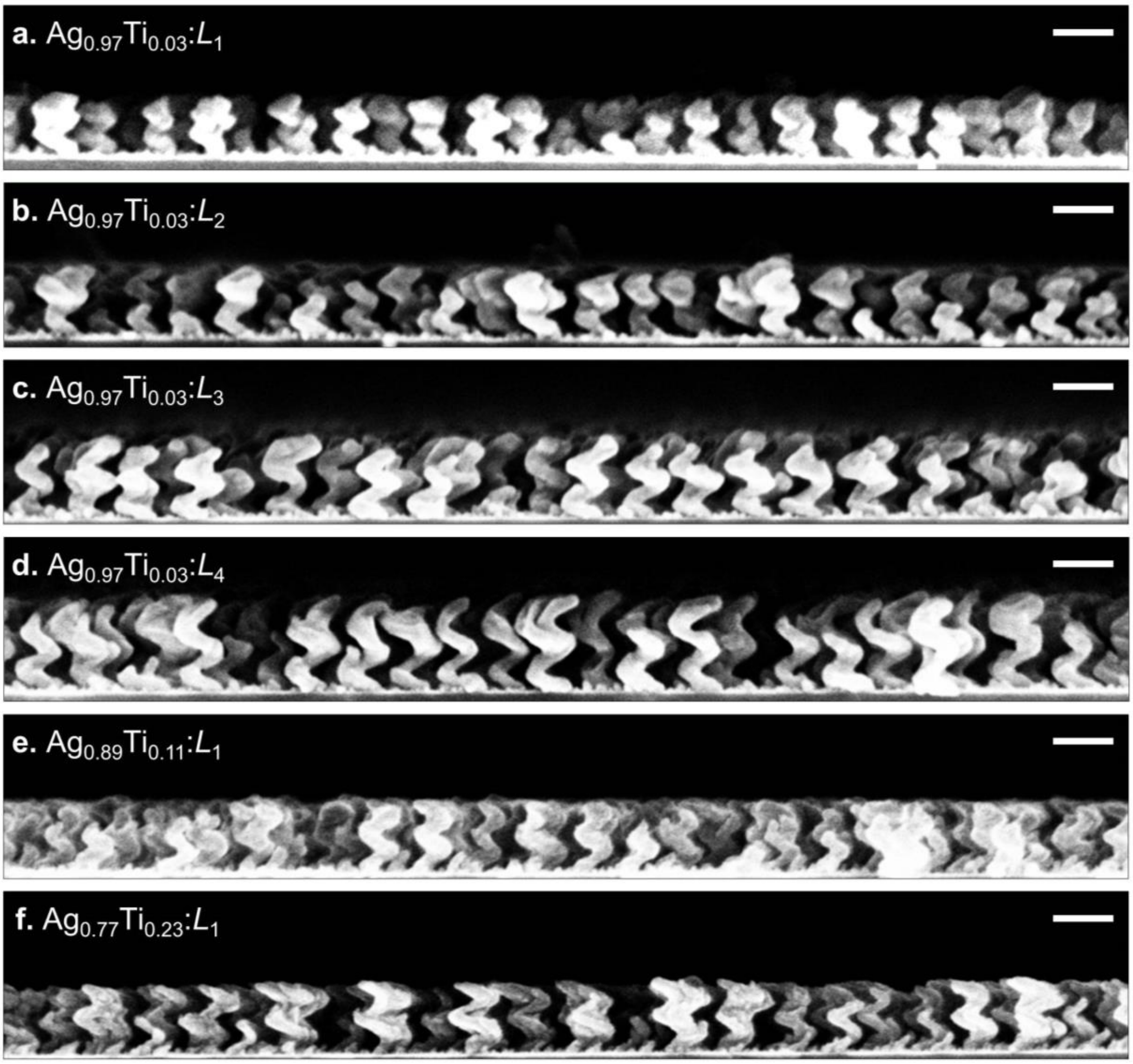

Supplementary Figure 1 The side view of SEM images of the grown Ag-Ti nanohelices. (a) $Ag_{0.97}Ti_{0.03}$:L_1, (b) $Ag_{0.97}Ti_{0.03}$:L_2, (c) $Ag_{0.97}Ti_{0.03}$:L_3, (d) $Ag_{0.97}Ti_{0.03}$:L_4, (e) $Ag_{0.89}Ti_{0.11}$:L_1, and (f) $Ag_{0.77}Ti_{0.23}$:L_1. (Scale bar: 100 nm).

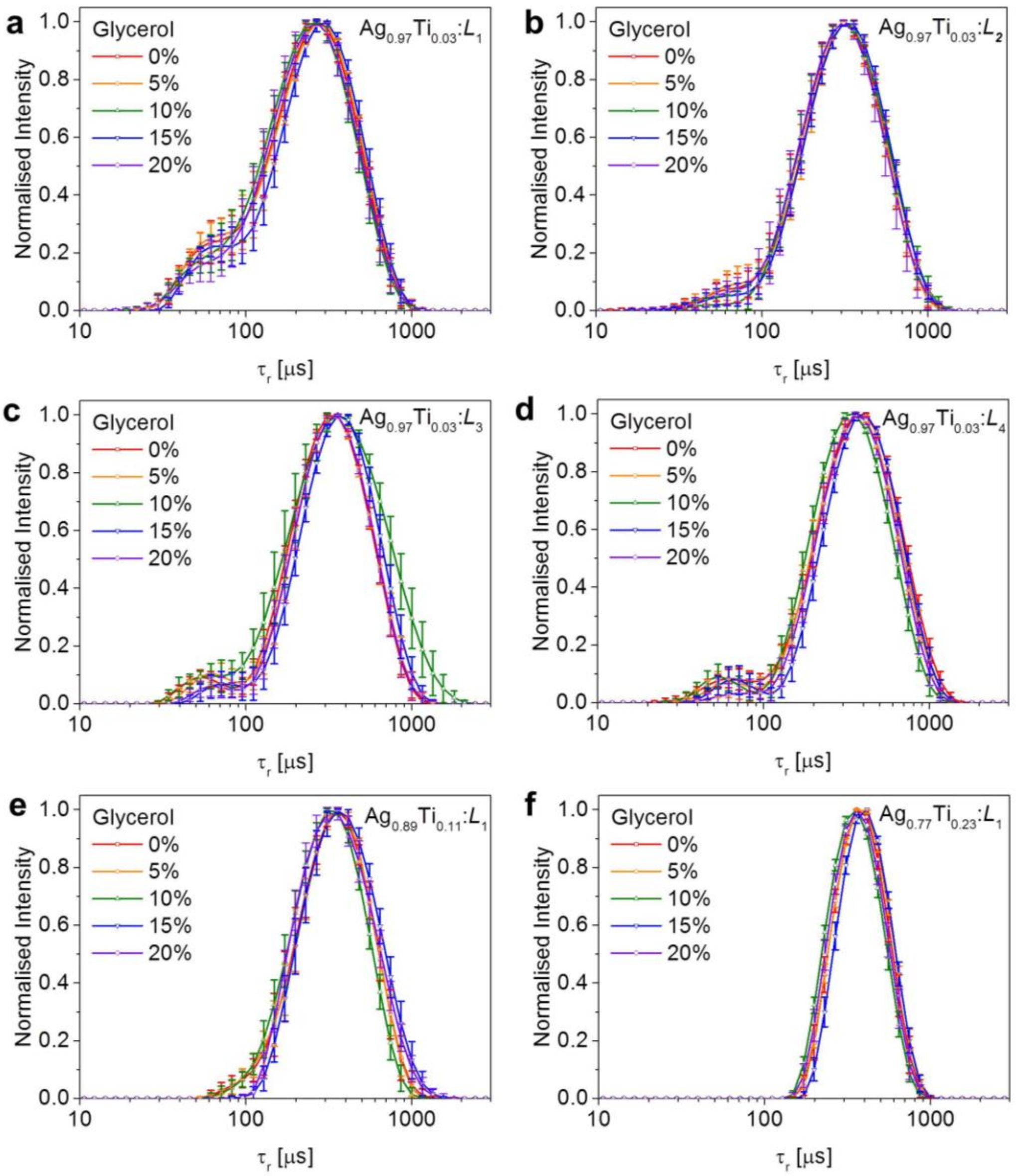

Supplementary Figure 2 Relaxation times of the colloidal Ag-Ti nanohelices in media of five different refractive indices (red: 0%, orange: 5%, green: 10%, blue: 15%, and violet: 20% glycerol/water mixtures) measured by the DLS. (a) $Ag_{0.97}Ti_{0.03}$:$L_{1,}$ (b) $Ag_{0.97}Ti_{0.03}$:L_2, (c) $Ag_{0.97}Ti_{0.03}$:L_3, (d) $Ag_{0.97}Ti_{0.03}$:L_4, (e) $Ag_{0.89}Ti_{0.11}$:L_1, and (f) $Ag_{0.77}Ti_{0.23}$:L_1.

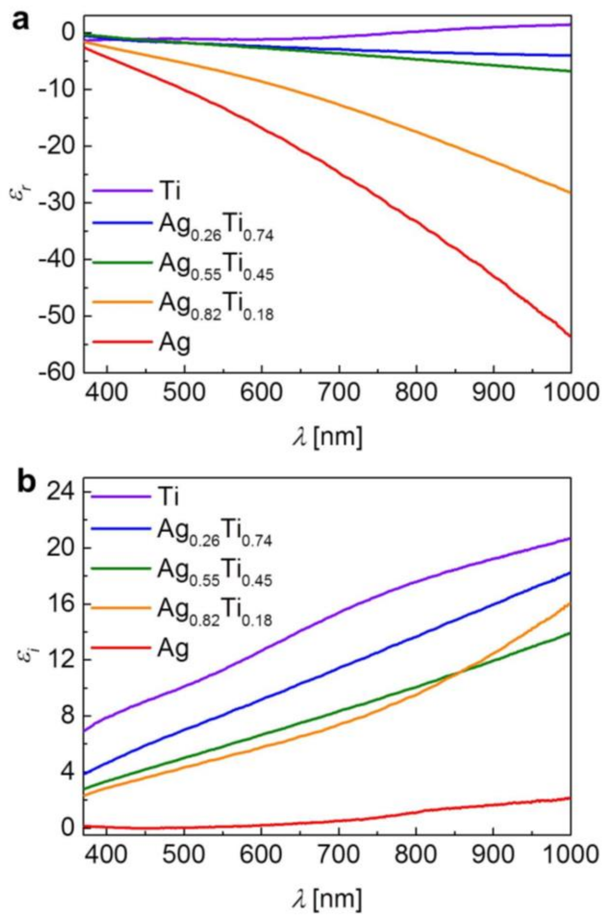

Supplementary Figure 3 The experimental (a) ε_r and (b) ε_i of Ag-Ti 150 nm thick films with different Ti contributions. (Ag: red, $Ag_{0.82}Ti_{0.18}$: orange, $Ag_{0.55}Ti_{0.45}$: green, $Ag_{0.26}Ti_{0.74}$: blue, and Ti: violet)

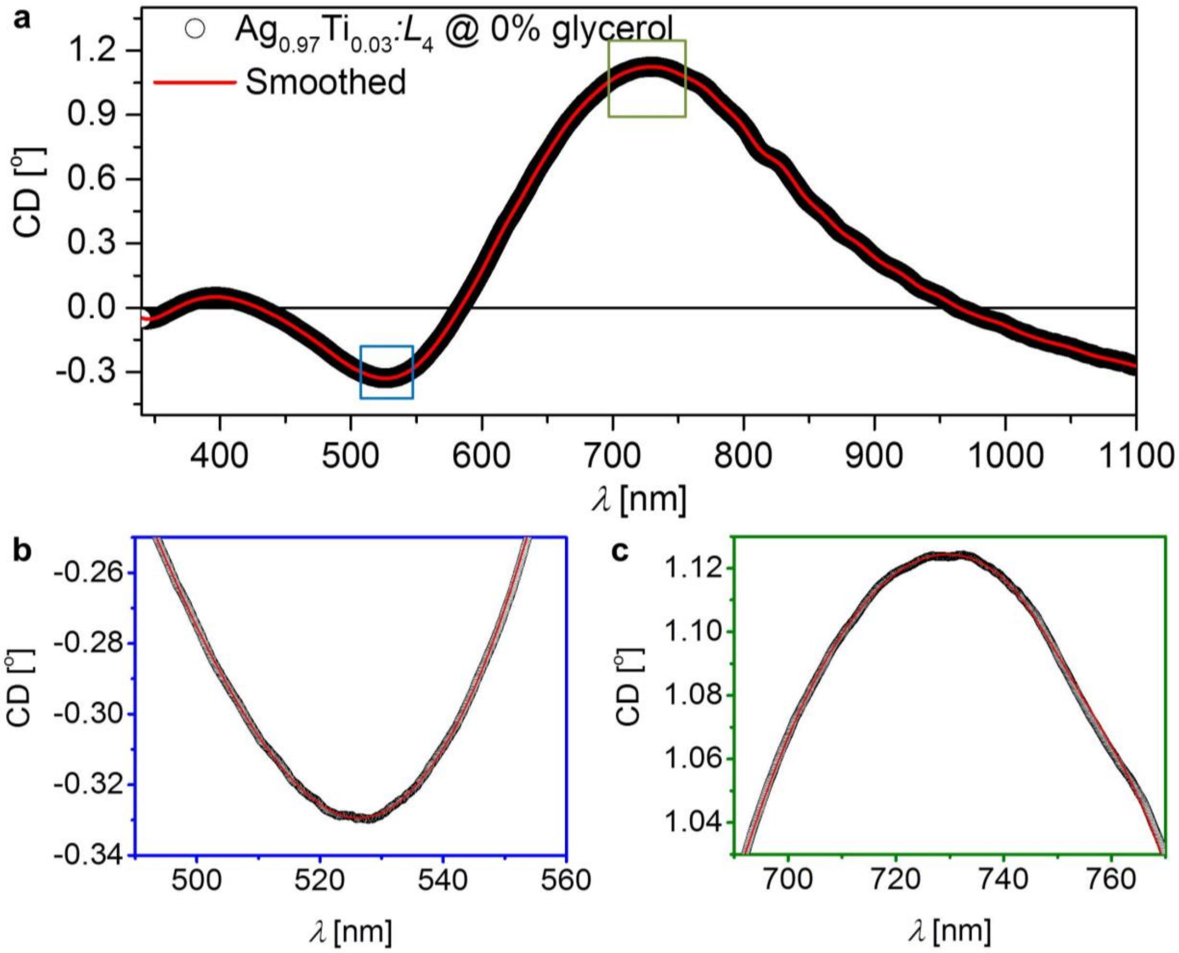

Supplementary Figure 4 Signal smoothing. (a) Comparison of the CD spectrum of colloidal $Ag_{0.97}Ti_{0.03}$:L_4 nanohelices at n = 1.333 RIU (water) before and after the signal smoothing process. (open black circle: measured data and red line: smoothed data). Its detailed plots of the resonance peaks at (b) λ_m and (c) λ_M.

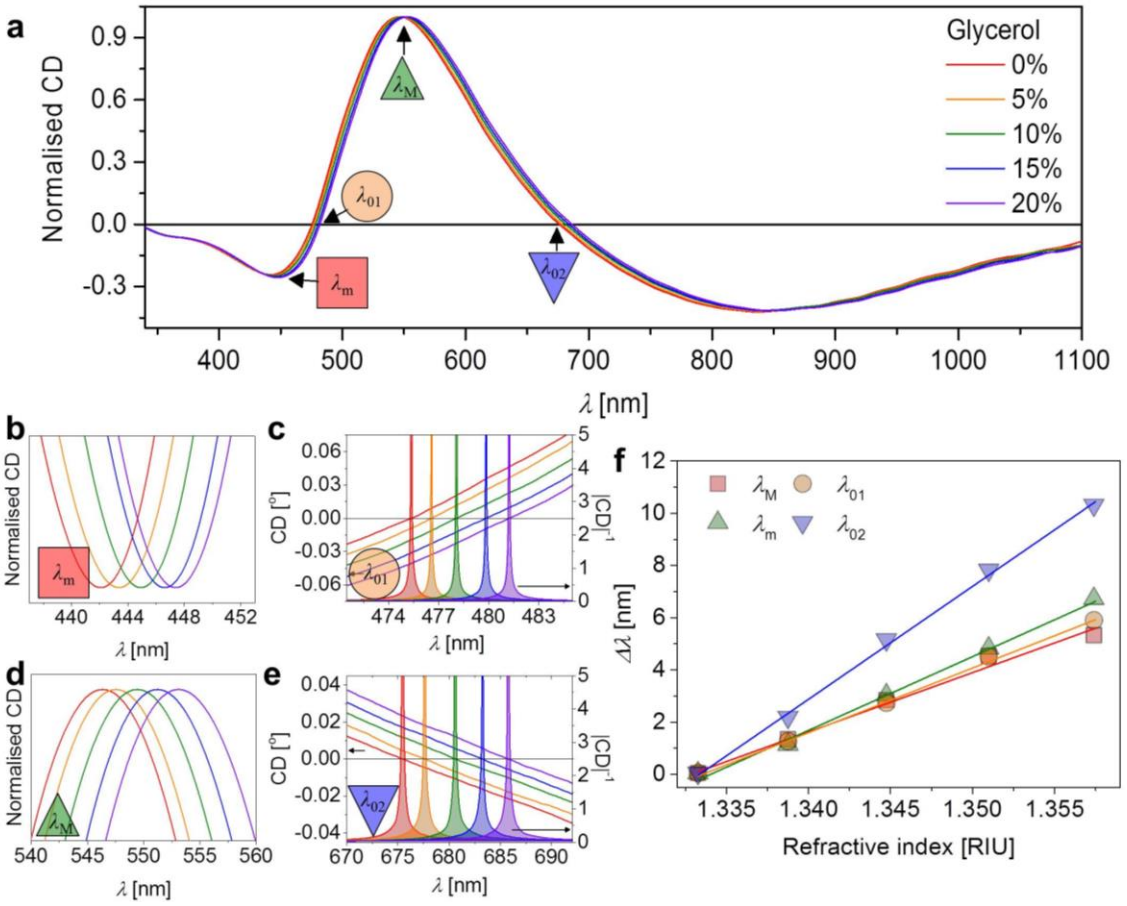

Supplementary Figure 5 (a) CD spectra of colloidal $Ag_{0.97}Ti_{0.03}$:L_1 nanohelices in media of five different refractive indices (red: 0%, orange: 5%, green: 10%, blue: 15%, and violet: 20% glycerol/water mixtures) over the full spectral range and detailed plots of the resonance shifts at (b) λ_{m} (red square), (c) λ_{01} (orange circle), (d) λ_{M} (green top triangle), and (e) λ_{02} (blue bottom triangle). (f) Wavelength shift, relative to water, of the four spectral features as a function of n.

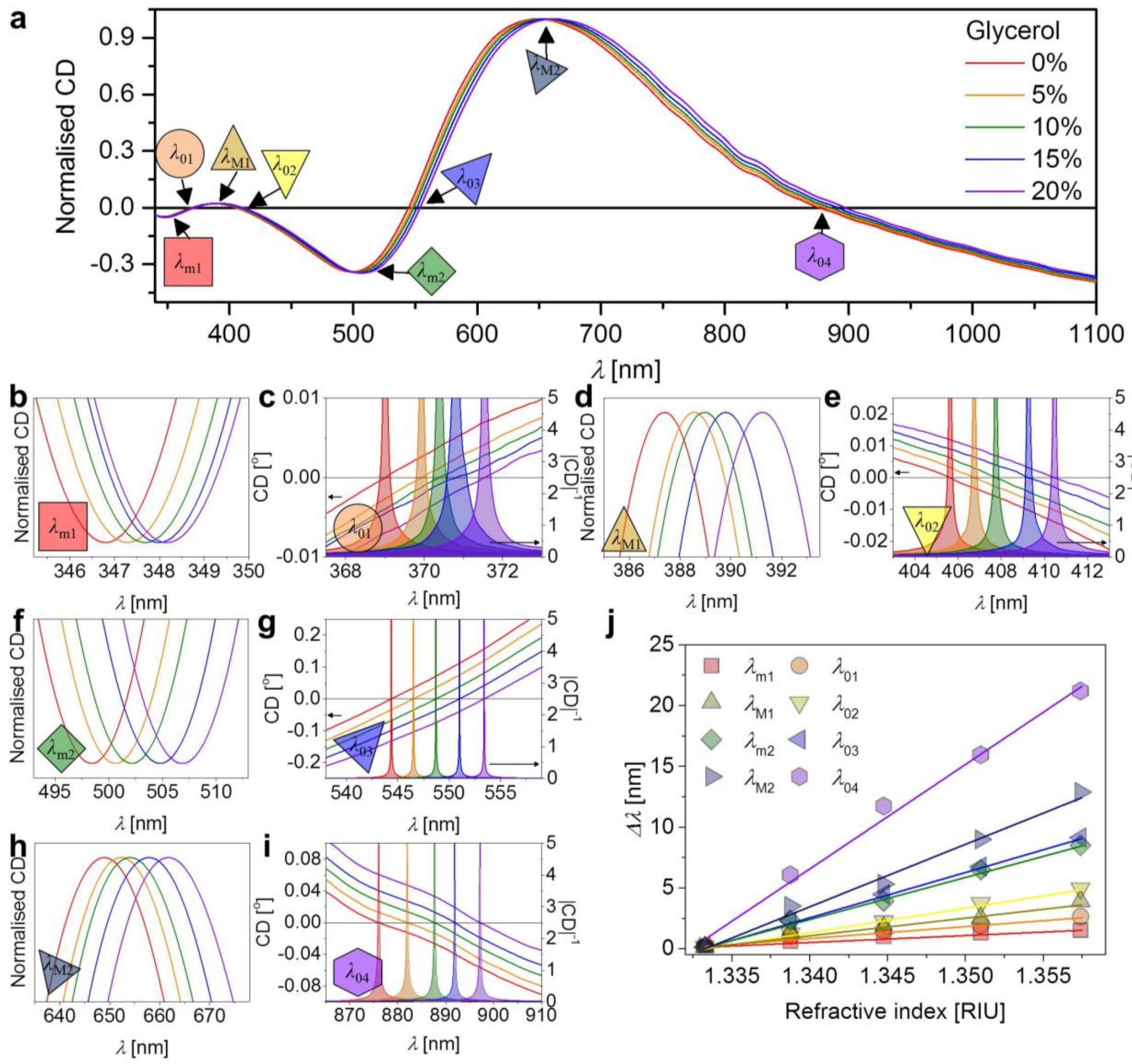

Supplementary Figure 6 (a) CD spectra of colloidal $Ag_{0.97}Ti_{0.03}$:L_3 nanohelices in media of five different refractive indices (red: 0%, orange: 5%, green: 10%, blue: 15%, and violet: 20% glycerol/water mixtures) over the full spectral range and detailed plots of the resonance shifts at (b) λ_{m1} (red square), (c) λ_{01} (orange circle), (d) λ_{M1} (dark yellow top triangle), (e) λ_{02} (yellow down triangle), (f) λ_{m2} (green diamond), (g) λ_{03} (blue left triangle), (h) λ_{M2} (navy right triangle), and (i) λ_{04} (violet hexagon). (j) Wavelength shift, relative to water, of the eight spectral features as a function of n.

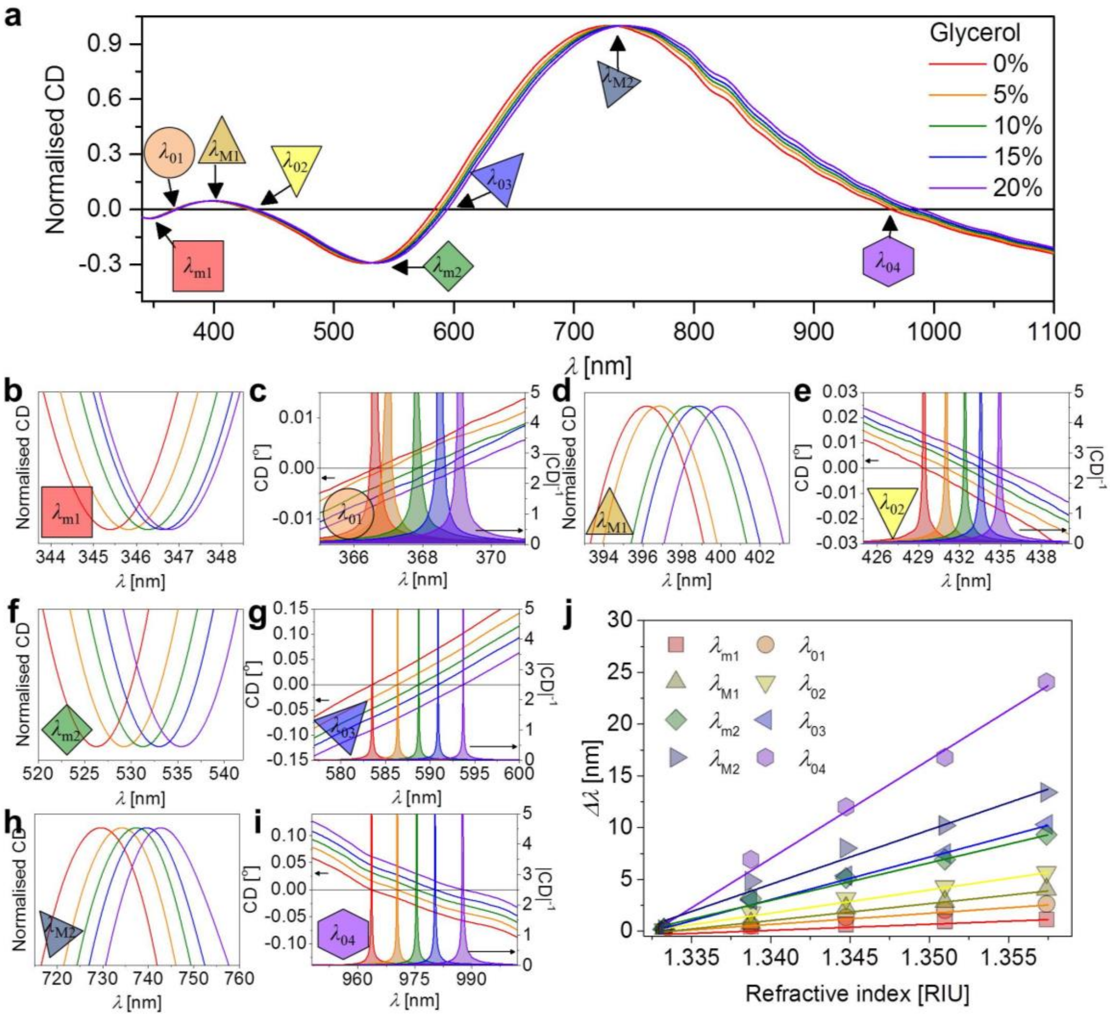

Supplementary Figure 7 (a) CD spectra of colloidal $Ag_{0.97}Ti_{0.03}$:L_4 nanohelices in media of five different refractive indices (red: 0%, orange: 5%, green: 10%, blue: 15%, and violet: 20% glycerol/water mixtures) over the full spectral range and detailed plots of the resonance shifts at (b) λ_{m1} (red square), (c) λ_{01} (orange circle), (d) λ_{M1} (dark yellow top triangle), (e) λ_{02} (yellow down triangle), (f) λ_{m2} (green diamond), (g) λ_{03} (blue left triangle), (h) λ_{M2} (navy right triangle), and (i) λ_{04} (violet hexagon). (j) Wavelength shift, relative to water, of the eight spectral features as a function of n.

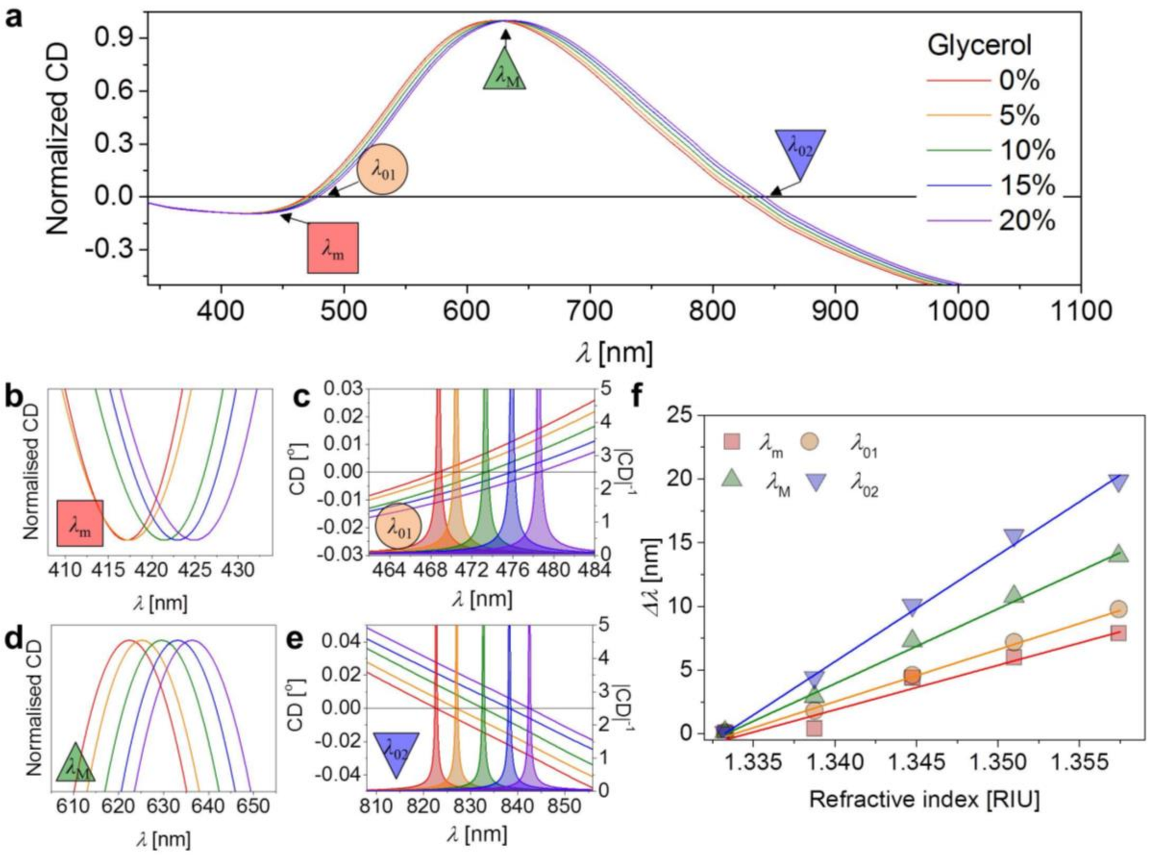

Supplementary Figure 8 (a) CD spectra of colloidal $Ag_{0.89}Ti_{0.11}$:L_1 nanohelices in media of five different refractive indices (red: 0%, orange: 5%, green: 10%, blue: 15%, and violet: 20% glycerol/water mixtures) over the full spectral range and detailed plots of the resonance shifts at (b) λ_{m} (red square), (c) λ_{01} (orange circle), (d) λ_{M} (green top triangle), and (e) λ_{02} (blue bottom triangle). (f) Wavelength shift, relative to water, of the four spectral features as a function of n.

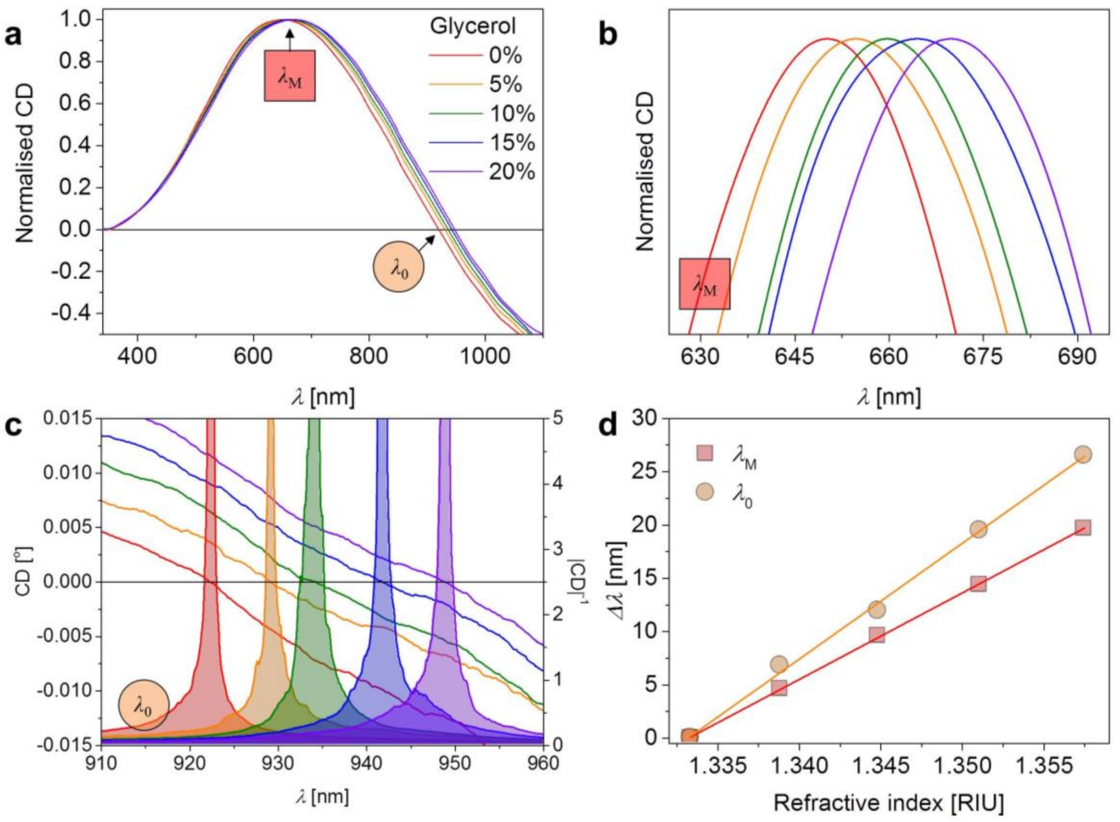

Supplementary Figure 9 (a) CD spectra of colloidal $Ag_{0.77}Ti_{0.23}$:L_1 nanohelices in media of five different refractive indices (red: 0%, orange: 5%, green: 10%, blue: 15%, and violet: 20% glycerol/water mixtures) over the full spectral range and detailed plots of the resonance shifts at (b) λ_M (red square) and (c) λ_0 (orange circle). (d) Wavelength shift, relative to water, of the two spectral features as a function of n.

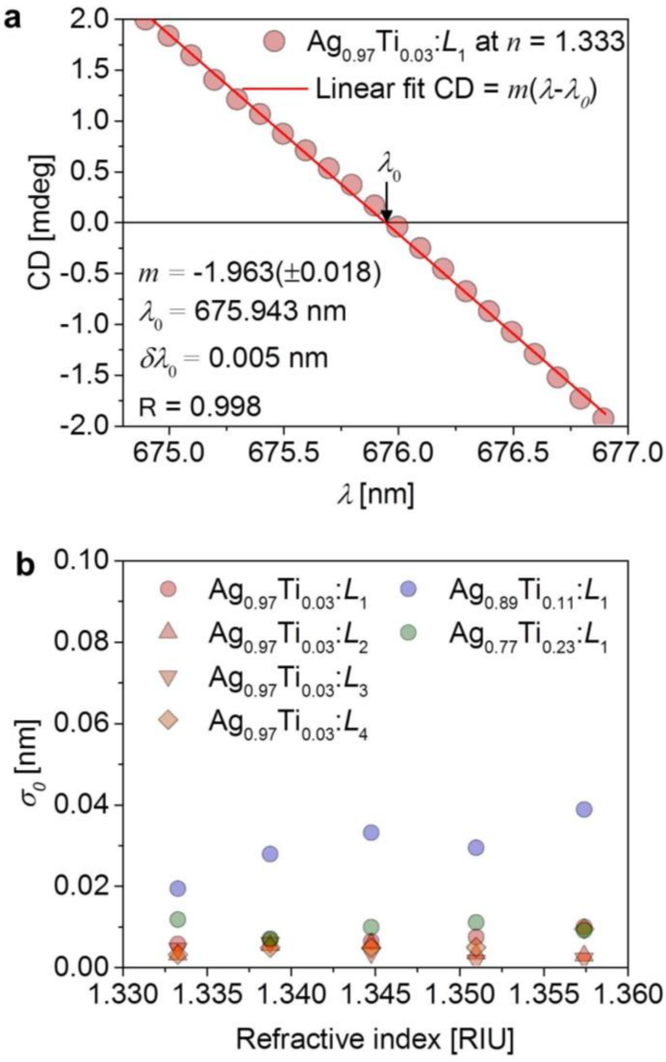

Supplementary Figure 10 (a) A CD spectrum of colloidal $Ag_{0.97}Ti_{0.03}$:L_1 nanohelices in water and its corresponding linear fit. (b) $\delta\lambda_0$ at λ_0 of $Ag_{0.97}Ti_{0.03}$:L_1 (red circle), $Ag_{0.97}Ti_{0.03}$:L_2 (red top-triangle), $Ag_{0.97}Ti_{0.03}$:L_3 (red bottom-triangle), $Ag_{0.97}Ti_{0.03}$:L_4 (red diamond), $Ag_{0.89}Ti_{0.11}$:L_1 (blue circle), and $Ag_{0.77}Ti_{0.23}$:L_1 (green circle) as a function of n.

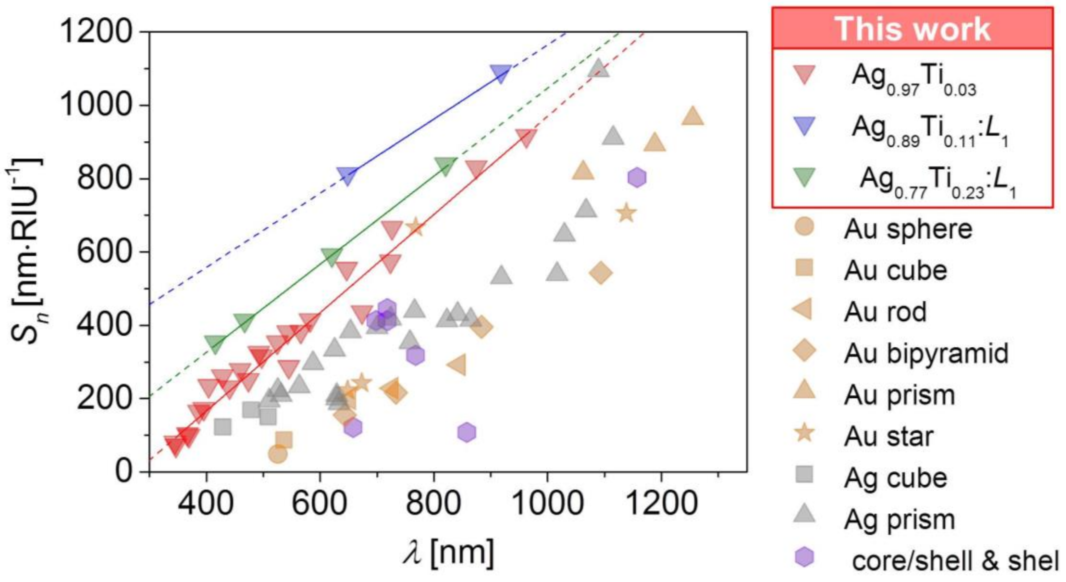

Supplementary Figure 11 The measured S_n of the Ag-Ti nanohelices (colour-filled symbols) and metallic nanocolloids (Au sphere[1], Au cube[1], Au rod[1], Au bipyramid[1], Au prism[2], Au star[1, 3, 4], Ag sphere[5, 6], Ag cube[7, 8], Ag prism[5, 9, 10], core/shell (or shell) [11, 12, 13, 14, 15]) as a function of λ at 1.333 RIU. The symbols and colours are different for the shapes and the material composition respectively.

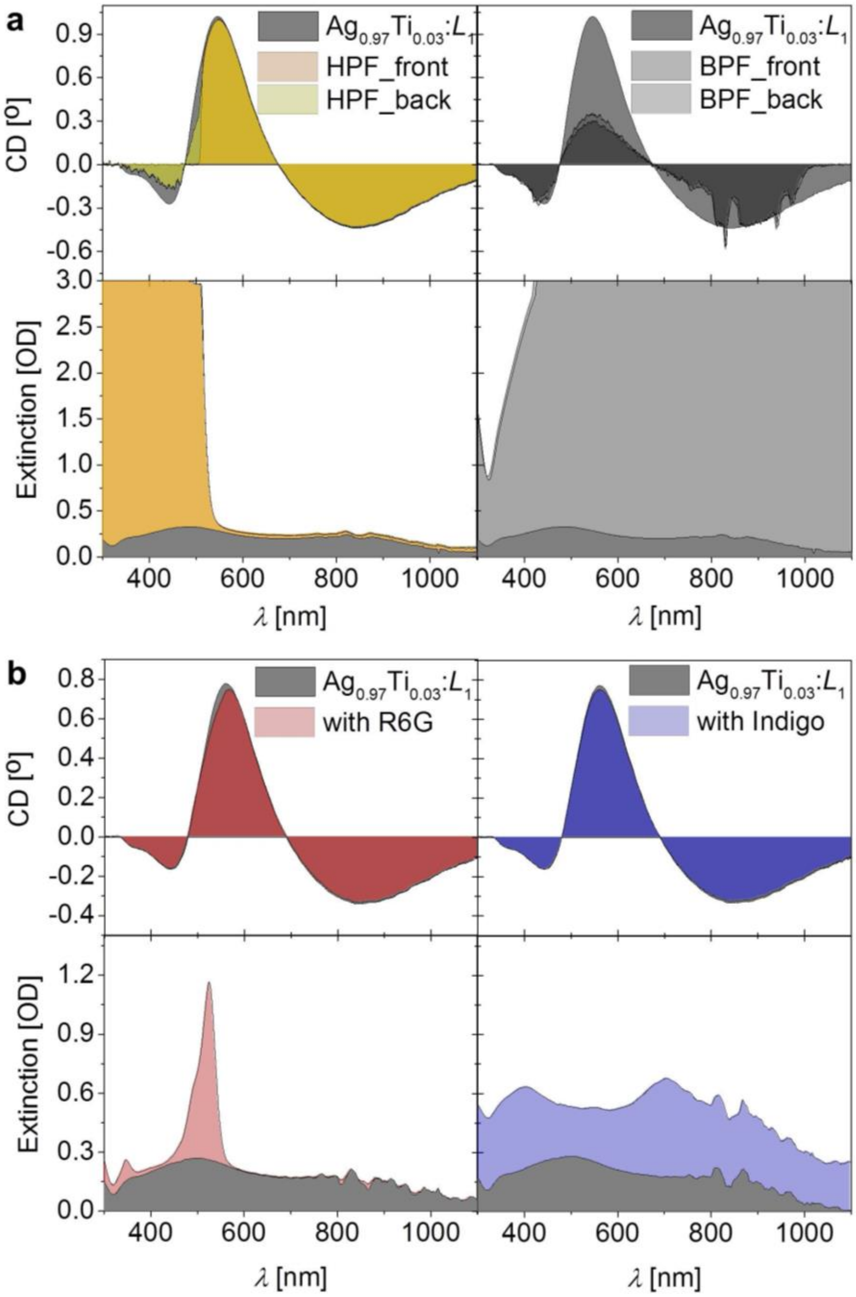

Supplementary Figure 12 CD spectra (each upper panel) and extinction spectra (each lower panel) of the colloidal $Ag_{0.97}Ti_{0.03}$:L_1 nanohelices in the presence of complex absorbing environments due to (a) optical filters (left panel: high pass, right panel: narrow band pass) and (b) molecular dyes (left panel: 10 µM rhodamine 6G, right panel: 100 µM indigo).

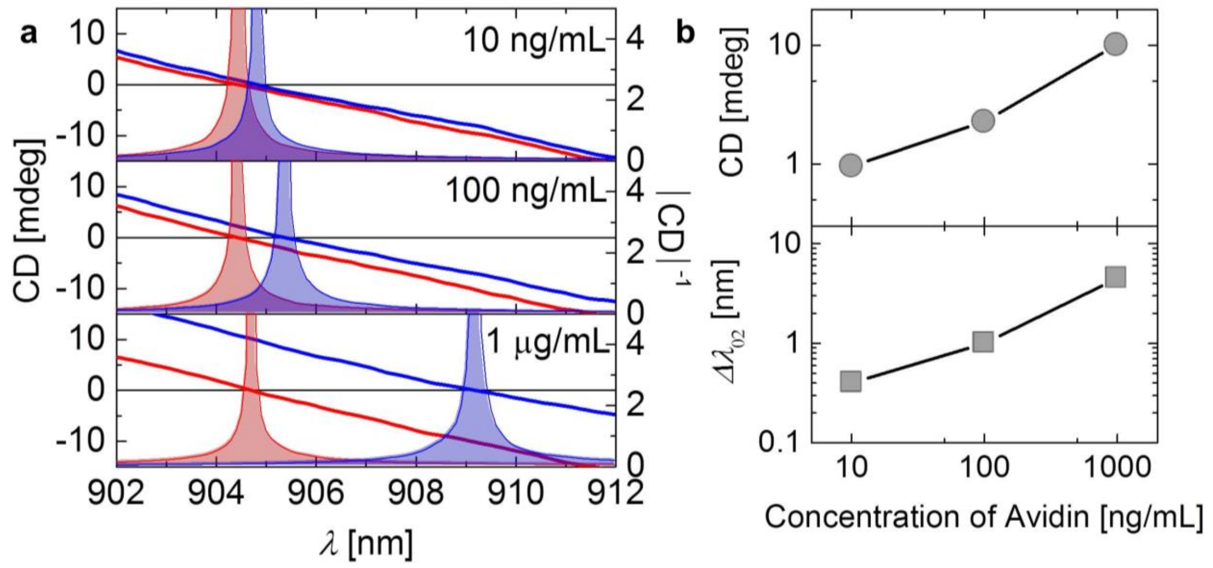

Supplementary Figure 13 Detection of biotin-avidin affinity for the biotin concentration ranging from 10 ng·mL^{-1} to 1 μg·mL^{-1}. (a) The resonance shift at λ_{02} of the colloidal $Ag_{0.89}Ti_{0.11}$:L_1 nanohelices for the different concentrations of avidin (10 ng·mL^{-1} to 1 μg·mL^{-1} with 1 order intervals, top to bottom). Each red signal and blue signal indicates the λ_{02} before and after avidin binding, respectively. (b) Change of CD at the initial λ_{02} (upper plot) and the wavelength shift $\Delta\lambda_{02}$ (lower plot) as a function of the injected concentrations of avidin.

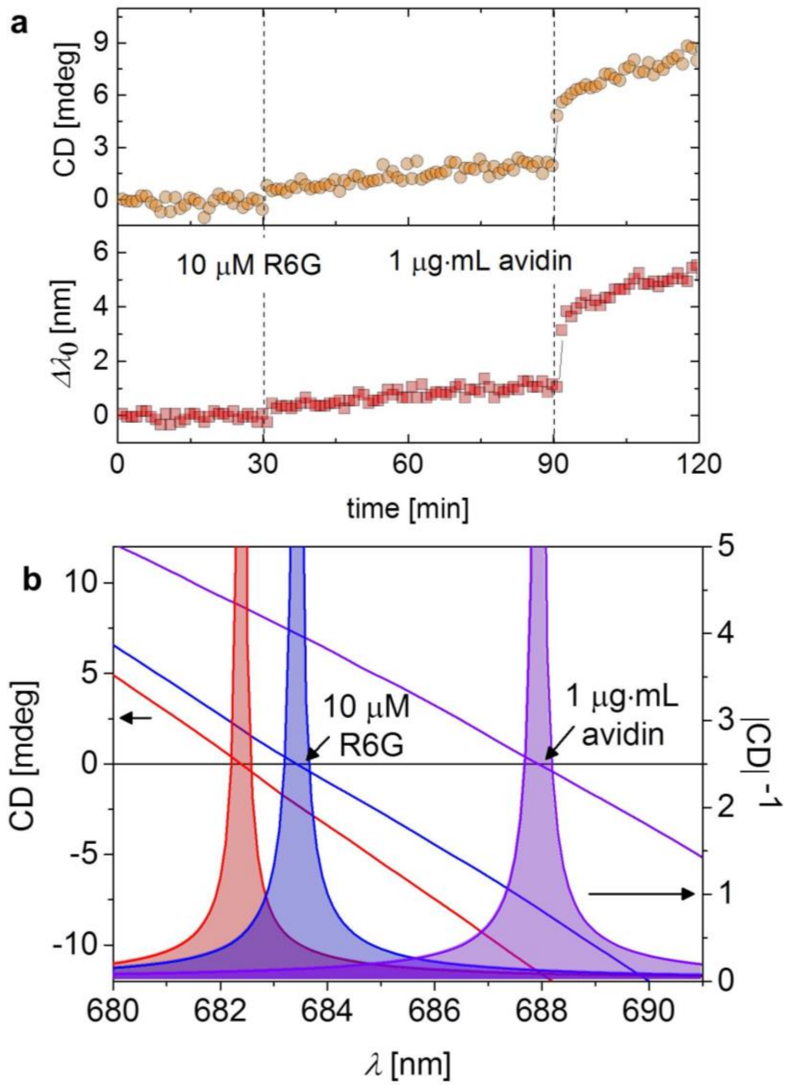

Supplementary Figure 14 Detection of biotin-avidin affinity in the presence of complex absorbing medium, 10 μM R6G. (a) *In-situ* measurements of the biotin-avidin interaction by monitoring the change of CD at the initial λ_{02} (upper plot) and the wavelength shift $\Delta\lambda_{02}$ (lower plot) with 1 min intervals. (b) Close-up view of the CD spectra for the biotinylated nanoparticle systems showing the wavelength shift and CD amplitude increase after R6G (blue) and avidin (violet) introduction.

Supplementary Table 1 Structural parameters of the grown Ag-Ti nanohelices

Sample	Atomic ratio of Ag : Ti [%]		Height [nm]	
	QCM	ICP-OES	QCM	SEM
$Ag_{0.97}Ti_{0.03}$:L_1	95 : 5	96.7 : 3.3 (±0.7)	600	113
$Ag_{0.97}Ti_{0.03}$:L_2	95 : 5	97.2 : 2.8 (±0.4)	700	128
$Ag_{0.97}Ti_{0.03}$:L_3	95 : 5	97.1 : 2.9 (±0.4)	800	141
$Ag_{0.97}Ti_{0.03}$:L_4	95 : 5	95.8 : 4.2 (±1.7)	900	157
$Ag_{0.89}Ti_{0.11}$:L_1	85 : 15	89.5 : 10.5 (±0.6)	600	119
$Ag_{0.77}Ti_{0.23}$:L_1	75 : 25	77.3 : 22.7 (±0.6)	600	124

Supplementary Table 2 ICP-OES measurements of the grown Ag-Ti thin films

Sample	Atomic ratio of Ag : Ti [%]
Ti	0 : 100 (±0)
$Ag_{0.26}Ti_{0.74}$	25.7 :74.3 (±0.3)
$Ag_{0.55}Ti_{0.45}$	54.6 : 45.4 (±0.7)
$Ag_{0.82}Ti_{0.18}$	82.3 : 17.7 (±0)
Ag	100 : 0 (±0)

Supplementary Note 1: Chiral plasmonic sensitivity, FWHM, and FOM

In order to evaluate the refractive index sensitivity (S_n), full width half maximum (FWHM), and figure of merit (FOM) of the chiral plasmonic sensing, we start by defining a chiral version of the plasmonic extinction formula

$$E_{\pm} \propto \frac{1}{\lambda}\left(\frac{\varepsilon_i}{(\varepsilon_r+(\overline{\chi}\pm\delta\chi)n^2)^2+\varepsilon_i^2}\right) \qquad (1)$$

Here, the shape factor has been split into an achiral component $\overline{\chi}$, and a part $\delta\chi$ which depends on the chirality of the particle. If the particle is achiral then $\delta\chi = 0$ and the equation above reduces to the usual one which peaks when $\varepsilon_r = -\overline{\chi}n^2$ [16, 17].

The circular dichroism is the difference between the extinctions for left and right handed polarised light

$$\mathrm{CD} \propto E_- - E_+ \qquad (2)$$

Nulls in the CD occur when

$$0 = E_- - E_+ \qquad (3)$$

$$\propto [\varepsilon_r + (\overline{\chi} - \delta\chi)n^2]^2 - [\varepsilon_r + (\overline{\chi} + \delta\chi)n^2]^2 \qquad (4)$$

$$= \delta\chi\varepsilon_r n^2 + \overline{\chi}\delta\chi n^4 \qquad (5)$$

$$\varepsilon_r^* = -\overline{\chi}n^2 \qquad (6)$$

So the CD crossing point corresponds to the peak position in the extinction of an achiral particle with the same $\overline{\chi}$. The wavelength of the resonance condition shifts with refractive index according to

$$S_n = \frac{d\lambda^*}{dn} = \frac{\frac{d\varepsilon_r^*}{dn}}{\left.\frac{d\varepsilon_r}{d\lambda}\right|_{\lambda^*}} = \frac{-2\overline{\chi}n}{\left.\frac{d\varepsilon_r}{d\lambda}\right|_{\lambda^*}} \qquad (7)$$

Near the crossing point the CD can be linearized to have a form something like $\mathrm{CD} = m(\lambda - \lambda^*)$. The reciprocal of the absolute value of this, $|CD|^{-1}$ is our "peak". Since it is divergent it has no well-defined maximum, so we follow the practice in Ref. [18], and define the maximum as the smallest CD we can reasonably measure: the instrumental resolution σ. This sets our peak maximum at σ^{-1}, which

means we're looking for the width at $\frac{1}{2}\sigma^{-1}$. This gives a half width at half max of $\lambda - \lambda^* = \frac{2\sigma}{|m|}$. So the full width is

$$\text{FWHM} = \frac{4\sigma}{|m|} = \frac{4\sigma}{\left|\frac{d\text{CD}}{d\lambda}\right|_{\lambda^*}} \tag{8}$$

What remains is to determine the slope. Let's start by looking at how the extinction for the (+) polarization changes with wavelength near λ^*.

$$\frac{dE_+}{d\lambda} = \frac{-E_+}{\lambda}\left(1 + \frac{2\lambda[\varepsilon_r+(\overline{\chi}+\delta\chi)n^2]}{[\varepsilon_r+(\overline{\chi}+\delta\chi)n^2]^2+\varepsilon_i^2}\frac{d\varepsilon_r}{d\lambda}\right) \tag{9}$$

The slope of the CD signal is the difference between the slopes for positive and negative polarizations.

$$\frac{d\text{CD}}{d\lambda} \propto \frac{dE_-}{d\lambda} - \frac{dE_+}{d\lambda} \tag{10}$$

$$= \frac{1}{\lambda}\left\{(E_- - E_+) + 2\lambda\left(\frac{\varepsilon_r+(\overline{\chi}-\delta\chi)n^2}{[\varepsilon_r+(\overline{\chi}-\delta\chi)n^2]^2+\varepsilon_i^2} - \frac{\varepsilon_r+(\overline{\chi}+\delta\chi)n^2}{[\varepsilon_r+(\overline{\chi}+\delta\chi)n^2]^2+\varepsilon_i^2}\right)\frac{d\varepsilon_r}{d\lambda}\right\} \tag{11}$$

At the crossing point, the first term (i.e. the CD) is, by definition, zero. Also, since at the crossing point the resonance condition is fulfilled, $\varepsilon_r + \overline{\chi}n^2 = 0$, and this reduces to

$$\left.\frac{d\text{CD}}{d\lambda}\right|_{\lambda^*} = \left(\frac{4\delta\chi n^2}{(\delta\chi n^2)^2+\varepsilon_i^2}\right)\left.\frac{d\varepsilon_r}{d\lambda}\right|_{\lambda^*} \tag{12}$$

$$= \left(\frac{4\delta\chi n^2}{\varepsilon_i^2}\right)\left.\frac{d\varepsilon_r}{d\lambda}\right|_{\lambda^*} \tag{13}$$

Combining this with Eqn. 8 gives

$$\text{FWHM} = \frac{4\sigma\varepsilon_i^2}{|\delta\chi|n^2\left|\frac{d\varepsilon_r}{d\lambda}\right|_{\lambda^*}} \tag{14}$$

and a figure of merit of

$$\text{FOM} = \frac{S_n}{\text{FWHM}} = \frac{|\delta\chi|n^2}{\sigma\varepsilon_i^2}\frac{d\varepsilon_r^*}{d\lambda} = \frac{2\overline{\chi}|\delta\chi|n^3}{\sigma\varepsilon_i^2} \tag{15}$$

Supplementary Note 2: Shape and material engineering of Ag-Ti nanohelices

The Ag-Ti nanohelices were grown by the shadow growth technique, namely nanoGLAD, developed by our group[19, 20]. The use of this technique permits controlling the geometrical sizes (pitch, line-width, and radius)[21, 22] as well as material composition of nanohelices[23]. Supplementary Table 1

presents all the information of the grown Ag-Ti nanohelices both that were programmed in the growth system and that were measured experimentally. Here, we have firstly controlled the sizes of the nanohelices with fixed material ratios of Ag : Ti = 97% : 3%, where Ag_xTi_y is the material atomic ratios of Ag and Ti with *x*% and *y*% respectively and L_n is the programmed length for the fabrications of the resultant nanohelices in the growth system. Next, at the given length L_1, we have tuned material composition ratios by controlling each material growth rate with closed-loop feedback to uniformly form the material composition through the whole nanostructure. The material composition was analysed by inductively coupled plasma optical emission spectroscopy (ICP-OES) and the measured values were considerably close to the desired values. Supplementary Fig. 1 shows the SEM images of the grown Ag-Ti nanohelices.

Supplementary Note 3: Colloidal stability

Considering the colloidal stability of the plasmonic nanoparticles is crucial as 'hotspot effect' from the closely neighbouring particles leads changing the absorption signals of the nanocolloids[24]. Hence, to prevent such disturbances on a linear function of the plasmonic resonance shift caused by the line-width broadening and peak drift, we have analysed the stability as well as the real *n* of the colloidal solutions of the Ag-Ti nanohelices by using dynamic light scattering (DLS)[25], during the CD measurement of specimen at the same time. Supplementary Fig. 2 shows the corresponding measured relaxation times of the colloids in 5 different refractive index media under the given material (Ag-refractive index: 0.135 and Absorption: 3.990) and environmental fitting parameter[26]. Such the overlapping responses over the 5 different dielectric media indicate that not only the prepared glycerol/water mixtures are well matched to the theoretical environmental conditions (e.g. viscosity, refractive index, etc.), but also the nanohelices have no significant aggregation in all the conditions.

Supplementary Note 4: Dielectric constants of Ag-Ti alloys

As a proof-of-concept experiment for the control of the dielectric constant over the whole wavelength range, we have grown 150 nm thickness of Ag-Ti bulk thin film on a 2 inch silicon wafer with different atomic ratios (Ti, $Ag_{0.26}Ti_{0.74}$, $Ag_{0.55}Ti_{0.45}$, $Ag_{0.82}Ti_{0.18}$, and Ag) and analysed their dielectric constants by the ellipsometry in the wavelength range of 300 nm to 1,100 nm. For the calculation of

the dielectric constants of such alloys, the effective medium approximation, assuming both the host and inclusion dielectric responses are already known, was required and we have used the Bruggeman model which is employed as[27]

$$n_{Ag}\left(\frac{\varepsilon_{Ag}-\varepsilon}{\varepsilon_{Ag}+2\varepsilon}\right)+n_{Ti}\left(\frac{\varepsilon_{Ti}-\varepsilon}{\varepsilon_{Ti}+2\varepsilon}\right)=0 \quad (16)$$

where n_{Ag} and n_{Ti} are the volume fractions of Ag and Ti, ε_{Ag} and ε_{Ti} are dielectric constants of Ag and Ti, and ε is effective dielectric constant of Ag-Ti alloy. The theoretical plots presented in Fig. 3a and b were also calculated by equation (16) with the Palik's ε_r of Ag and Ti[28]. Supplementary Fig. 3 shows the dielectric constants of the grown Ag-Ti thin films having different atomic ratios and this clearly presents that the dielectric constants of materials can experimentally be tuned by mixing two (or more) materials as expected theoretically.

Supplementary Note 5: Bulk refractive index sensing

To clarify the peak positions at λ_{M} and λ_{m} from the small signal fluctuation caused by the mechanical damping of the gratings, we have smoothed the measured CD spectrum in the Origin software. Supplementary Fig. 4 shows, as an example, the comparison of CD spectrum of colloidal $Ag_{0.97}Ti_{0.03}$:L_4 nanohelices before and after signal smoothing process and this presents that the smoothed signal helps to recognise the peaks, but does not distort the signal information. The following Supplementary Fig. 5-9 show the bulk refractive index sensing using different colloidal solutions of the grown Ag-Ti nanohelices, $Ag_{0.97}Ti_{0.03}$:L_1, $Ag_{0.97}Ti_{0.03}$:L_3, $Ag_{0.97}Ti_{0.03}$:L_4, $Ag_{0.89}Ti_{0.11}$:L_1, and $Ag_{0.77}Ti_{0.23}$:L_1 respectively.

Supplementary Note 6: Spectroscopic resolution at zero-crossing

We use a simple linear fit of the form $\mathrm{CD} = m(\lambda - \lambda_0)$ to fit the bipolar CD signal near the zero crossing point λ_0. The standard error of the fit parameter, $\delta\lambda_0$ is indicative of the precision with which the crossing point can be localized[18]. Supplementary Fig. 10a shows an example of the data analysis with the CD spectrum of colloidal $Ag_{0.97}Ti_{0.03}$:L_1 nanohelices in water. In this case the resolution, $\delta\lambda_0$ was 0.005 nm. Supplementary Fig. 10b shows $\delta\lambda_0$ as a function of n taken from the CD spectra of all the Ag-Ti nanohelices.

Supplementary Note 7: Comparison of LSPR sensitivities of metallic nanocolloids

In order to visualize the sensing performance of our dispersion and shape engineered Ag-Ti nanohelices, we have summarised those bulk refractive index sensitivities in comparison with the sensitivities reported to date in literature for metallic nanocolloids having various different shape and material composition in Supplementary Fig. 11.

Supplementary Note 8: Optical response in the presence of complex absorbers

We use a colloidal suspension of $Ag_{0.97}Ti_{0.03}$:L_1 nanohelices in water with the addition of absorbers in the form of low-pass (left) and narrowband (right) filters in the optical path where the maximum absorbance exceeds an optical density of 3 (<0.1% transmission, lower panels), Supplementary Fig. 12a. As expected the CD signals are still measureable even if small distortions are introduced. The same experiment demonstrated with molecular absorbers rhodamine 6G (left) and indigo (right) yield similar results, Supplementary Fig. 12b (see Fig. 5 for sensing performance in the presence of complex absorbing media).

Supplementary Note 9: Biotin-avidin affinity sensing

Biotin-avidin affinity is a well-known common binding protocol and this is often used as a proof-of-concept experiment for plasmonic biosensors[29]. We have also used this to describe how our scheme can be used for bio-sensing in order to detect the small weight change near the surface of the particles (Fig. 5). We first immobilise the biotins on the surface of $Ag_{0.89}Ti_{0.11}$:L_1 nanohelices by the thiol (SH-) coupling overnight. Next, we measure the CD spectra of such colloids before introducing the avidin into them, then we add avidin with a controlled concentration ranging from 10 $ng \cdot mL^{-1}$ to 1 $\mu g \cdot mL^{-1}$ with 1 order increment. After 1 h we measure their CD spectra again, Supplementary Fig. 13. As expected, this shows the differential shifts as a function of the introduced avidin concentrations. Moreover, this binding was clearly monitored even under the complex absorbing medium, 10 μM R6G, Supplementary Fig. 14.

Supplementary References

1. Chen H, Kou X, Yang Z, Ni W, Wang J. Shape- and Size-Dependent Refractive Index Sensitivity of Gold Nanoparticles. *Langmuir* **24**, 5233-5237 (2008).

2. Liu R, *et al.* On-demand shape and size purification of nanoparticle based on surface area. *Nanoscale* **6**, 13145-13153 (2014).

3. Nehl CL, Liao H, Hafner JH. Optical Properties of Star-Shaped Gold Nanoparticles. *Nano Letters* **6**, 683-688 (2006).

4. Dondapati SK, Sau TK, Hrelescu C, Klar TA, Stefani FD, Feldmann J. Label-free Biosensing Based on Single Gold Nanostars as Plasmonic Transducers. *ACS Nano* **4**, 6318-6322 (2010).

5. Mock JJ, Smith DR, Schultz S. Local Refractive Index Dependence of Plasmon Resonance Spectra from Individual Nanoparticles. *Nano Letters* **3**, 485-491 (2003).

6. McFarland AD, Van Duyne RP. Single Silver Nanoparticles as Real-Time Optical Sensors with Zeptomole Sensitivity. *Nano Letters* **3**, 1057-1062 (2003).

7. Sherry LJ, Chang S-H, Schatz GC, Van Duyne RP, Wiley BJ, Xia Y. Localized Surface Plasmon Resonance Spectroscopy of Single Silver Nanocubes. *Nano Letters* **5**, 2034-2038 (2005).

8. Galush WJ, Shelby SA, Mulvihill MJ, Tao A, Yang P, Groves JT. A Nanocube Plasmonic Sensor for Molecular Binding on Membrane Surfaces. *Nano Letters* **9**, 2077-2082 (2009).

9. Charles DE, *et al.* Versatile Solution Phase Triangular Silver Nanoplates for Highly Sensitive Plasmon Resonance Sensing. *ACS Nano* **4**, 55-64 (2010).

10. Sherry LJ, Jin R, Mirkin CA, Schatz GC, Van Duyne RP. Localized Surface Plasmon Resonance Spectroscopy of Single Silver Triangular Nanoprisms. *Nano Letters* **6**, 2060-2065 (2006).

11. Tam F, Moran C, Halas N. Geometrical Parameters Controlling Sensitivity of Nanoshell Plasmon Resonances to Changes in Dielectric Environment. *The Journal of Physical Chemistry B* **108**, 17290-17294 (2004).

12. Zhou N, Ye C, Polavarapu L, Xu Q-H. Controlled preparation of Au/Ag/SnO2 core-shell nanoparticles using a photochemical method and applications in LSPR based sensing. *Nanoscale* **7**, 9025-9032 (2015).

13. Sun Y, Xia Y. Increased Sensitivity of Surface Plasmon Resonance of Gold Nanoshells Compared to That of Gold Solid Colloids in Response to Environmental Changes. *Analytical Chemistry* **74**, 5297-5305 (2002).

14. Wang H, Brandl DW, Le F, Nordlander P, Halas NJ. Nanorice: A Hybrid Plasmonic Nanostructure. *Nano Letters* **6**, 827-832 (2006).

15. Raschke G, *et al.* Gold Nanoshells Improve Single Nanoparticle Molecular Sensors. *Nano Letters* **4**, 1853-1857 (2004).

16. Miller MM, Lazarides AA. Sensitivity of Metal Nanoparticle Surface Plasmon Resonance to the Dielectric Environment. *The Journal of Physical Chemistry B* **109**, 21556-21565 (2005).

17. Lee K-S, El-Sayed MA. Gold and Silver Nanoparticles in Sensing and Imaging: Sensitivity of Plasmon Response to Size, Shape, and Metal Composition. *The Journal of Physical Chemistry B* **110**, 19220-19225 (2006).

18. Maccaferri N, *et al.* Ultrasensitive and label-free molecular-level detection enabled by light phase control in magnetoplasmonic nanoantennas. *Nat Commun* **6**, (2015).

19. Mark AG, Gibbs JG, Lee T-C, Fischer P. Hybrid nanocolloids with programmed three-dimensional shape and material composition. *Nat Mater* **12**, 802-807 (2013).

20. Jeong H-H, *et al.* Selectable Nanopattern Arrays for Nanolithographic Imprint and Etch-Mask Applications. *Advanced Science* **2**, n/a-n/a (2015).

21. Gibbs JG, Mark AG, Eslami S, Fischer P. Plasmonic nanohelix metamaterials with tailorable giant circular dichroism. *Applied Physics Letters* **103**, 213101 (2013).

22. Gibbs JG, Mark AG, Lee T-C, Eslami S, Schamel D, Fischer P. Nanohelices by shadow growth. *Nanoscale* **6**, 9457-9466 (2014).

23. Eslami S, *et al.* Chiral Nanomagnets. *ACS Photonics* **1**, 1231-1236 (2014).

24. de la Rica R, Stevens MM. Plasmonic ELISA for the ultrasensitive detection of disease biomarkers with the naked eye. *Nat Nano* **7**, 821-824 (2012).

25. Lee T-C, Alarcón-Correa M, Miksch C, Hahn K, Gibbs JG, Fischer P. Self-Propelling Nanomotors in the Presence of Strong Brownian Forces. *Nano Letters* **14**, 2407-2412 (2014).

26. Cheng N-S. Formula for the Viscosity of a Glycerol−Water Mixture. *Industrial & Engineering Chemistry Research* **47**, 3285-3288 (2008).

27. Choy TC. *Effective Medium Theory: Principles and Applications*. Clarendon Press (1999).

28. Palik ED. *Handbook of optical constants of solids*. Academic Press (1985).

29. Kabashin AV, *et al.* Plasmonic nanorod metamaterials for biosensing. *Nat Mater* **8**, 867-871 (2009).
